# Supplementary material for: Assessing Smoke-Free Housing Implementation Approaches to Inform Best Practices: A National Survey of Early-Adopting Public Housing Authorities
Source: Int J Environ Res Public Health. 2022 Mar 24;19(7):3854. doi: 10.3390/ijerph19073854 (PMC8997519; doi:10.3390/ijerph19073854)
Supplement: Supplementary file 1 [file ijerph-19-03854-s001.zip › ijerph-1621580-supplementary.pdf]

Please complete this survey to the best of your ability. If you are unsure how to answer a question, please do not guess. Rather, use the 'don't know' option.

Please respond to each question. Scroll down each page of the survey to respond to all questions on that page.

Use the << >> buttons to navigate backwards or forwards through pages to view or edit your responses. Do not use the back/forward buttons on your browser to attempt to navigate through the survey (your responses will not be saved).

Throughout the survey we will distinguish between the:
1) planning and preparation phase which includes activities leading up to the date of your smoke-free policy adoption and,
2) implementation phase which are activities after the date of your policy adoption. Included in each of these phases are tasks such as engagement, enforcement, training, cessation counseling, and tracking/monitoring.

Which of the following best describes your role at this PHA:

- ○ Executive Director
- ○ Property Manager
- ○ Housing Authority Administrator
- ○ Resident Service Coordinator
- ○ Facilities Director
- ○ Other (please specify): ______________________________

How long have you worked at this PHA?

- ○ Less than 6 months
- ○ Between 6 months and 1 year
- ○ Between 1- 3 years
- ○ 3-5 years
- ○ 6-10 years
- ○ More than 10 years

Were you at this PHA during the *planning and preparation phase* of the smoke-free policy (ie. before adoption)?

- ○ Yes
- ○ No

How involved were you in the *planning and preparation* of the smoke-free policy?

- ○ To a great extent
- ○ Somewhat
- ○ Very little
- ○ Not at all
- ○ Don't know

How involved are you in the ongoing *implementation* of the smoke-free policy (ie, post-adoption)?

- ○ To a great extent
- ○ Somewhat
- ○ Very Little
- ○ Not at all
- ○ Don't know

In what year was your smoke-free policy adopted?

____

Does your smoke-free policy prohibit smoking *everywhere* on the property (indoors and outdoors)?

- ○ Yes
- ○ No
- ○ Don't know

If no, please select ALL of the places on the properties where people are permitted to smoke (Check all that apply):

- ☐ Inside individual units
- ☐ Individual unit balconies, patios or porches
- ☐ Designated indoor smoking area
- ☐ Designated outdoor smoking area
- ☐ Beyond a defined buffer zone from building entrances and doorways -- If marked, what is the number of feet of the buffer zone): ______________________________
- ☐ Other (please specify): ______________________________

Are there any special accommodations for individual residents provided within your no-smoking policy (e.g. for mobility impairment)?

- ○ Yes
- ○ No
- ○ Don't know

What are those accommodations? Select all that apply.

- ☐ Moving the smoker to a different apartment so it is easier to get outside
- ☐ Making changes to help the smoker go outside (e.g. ramp)
- ☐ Designated indoor smoking area
- ☐ Allowing a person to smoke in their apartment
- ☐ Other: please specify ______________________________

Does your policy prohibit or allow the use of e-cigarettes?

- ◯ Prohibit
- ◯ Allow
- ◯ Other (please specify) ______________________________
- ◯ Don't know

Next we'd like to ask about how residents may have been engaged before and after the adoption of the smoke-free policy.

During the *preparation and planning phase* (i.e., *before* the policy was adopted), select which of the following activities your PHA conducted and how helpful they were in gaining acceptance of the policy?

	Did you do this activity?			If used, how helpful was it?					
	Yes	No	Don't Know	Un-helpful	Some-what Un-helpful	Neither helpful or un-helpful	Some-what helpful	Help-ful	Don't Know
Survey residents to gauge support and opposition to the policy									
Host resident information sessions about the policy									
Include residents in decision-making committees about whether to adopt the policy									
Other (please specify):									

In what ways did residents help decide key aspects of the no-smoking policy BEFORE it went into place, and how helpful was such participation in gaining acceptance of the policy?

	Did you do this activity?			If used, how helpful was it?					
	Yes	No	Don't Know	Un-helpful	Somewhat Unhelpful	Neither helpful or unhelpful	Some-what helpful	Helpful	Don't Know
Where smoking is not permitted									
Enforcement processes									
Cessation support activities									
Inclusion/exclusion of e-cigarettes									
Other (please specify):									

What was the level of overall SUPPORT of the policy among residents before it was adopted?

- ○ Mostly unsupportive
- ○ Somewhat unsupportive
- ○ Neither unsupportive or supportive
- ○ Somewhat supportive
- ○ Mostly supportive
- ○ Don't know

What is the level of overall SUPPORT of the policy among residents now?

- ○ Mostly unsupportive
- ○ Somewhat unsupportive
- ○ Neither unsupportive or supportive
- ○ Somewhat supportive
- ○ Mostly supportive
- ○ Don't know

Now thinking about *ON-GOING IMPLEMENTATION (i.e., after adoption of the policy),* do residents participate in the following on-going adherence activities (check all that apply):

- ☐ Enforcement Processes
- ☐ Cessation Support Activities
- ☐ Other (please specify): ______________________________
- ☐ None
- ☐ Don't know

Next, we would like to learn more about the types of tobacco cessation support you may have offered to PHA residents as part of your PHA's smoke-free policy.

Please check all of the smoking cessation support activities you offered AT ANY POINT during the planning and preparation phase or after implementation.

	Is this mechanism offered?		
	Yes	No	Don't Know
On-site smoking cessation counseling			
Referrals to smoking cessation counseling			
Quit line number or information about other resources			
Nicotine replacement therapy (e.g. patches, gum, lozenges)			
Quit smoking information (e.g. booklet, brochure, video, etc.)			
Suggestions to contact primary care provider			
Other (please specify):			

Which categories of development staff have received training about the smoke-free policy? Check all that apply.

- ☐ Property Manager
- ☐ Resident Service Coordinator
- ☐ Maintenance Staff
- ☐ Security Staff
- ☐ Asthma or other Community Health Workers
- ☐ Others (please specify) ____

In what ways have staff been trained to implement the policy, and how helpful has each been?

	Was there training for this?			If used, how helpful was it?					
	Yes	No	Don't Know	Unhelpful	Somewhat Unhelpful	Neither helpful or unhelpful	Somewhat helpful	Helpful	Don't Know
General information about the smoke-free policy (e.g. where people can and can't smoke)									
Effects of second hand smoke exposure									
Resident outreach and engagement									
Procedures for identifying violations									
Procedural mechanisms for enforcement/ responding to violations									
Basic skills on smoking cessation									
Resources available for helping smokers quit									
General communication and negotiation skills									
Other (please specify):									

How challenging did staff find the preparation and planning for the smoke-free policy?

- ◯ To a great extent
- ◯ Somewhat
- ◯ Very Little
- ◯ Not at all
- ◯ Don't know

What was the level of overall SUPPORT of the policy among staff before it was adopted?

- ◯ Mostly unsupportive
- ◯ Somewhat unsupportive
- ◯ Neither unsupportive nor supportive
- ◯ Somewhat supportive
- ◯ Mostly supportive
- ◯ Don't know

What is the level of overall SUPPORT of the policy among staff now?

- ◯ Mostly unsupportive
- ◯ Somewhat unsupportive
- ◯ Neither unsupportive nor supportive
- ◯ Somewhat supportive
- ◯ Mostly supportive
- ◯ Don't know

Have you developed partnerships with any of the following community organizations to plan or implement the smoke-free policy? Check all that apply.

- ☐ Community Health Centers
- ☐ Local Hospitals
- ☐ Public Health Departments
- ☐ Community Service Agencies
- ☐ Residence Advocacy Groups
- ☐ Voluntary Organizations (e.g. American Lung Association)
- ☐ Academic Groups or Organizations
- ☐ Other (Please Specify): ______________________________

Did any of these partnerships provide funding or services to support your smoke-free policy?

- ○ Yes, please explain: ______________________________
- ○ No
- ○ Don't Know

Next we would like to learn about the processes and procedures in place for enforcement.

Has the smoke-free policy been incorporated into the lease?

- ◯ Yes
- ◯ No
- ◯ Don't know

Among known violations to the smoke-free policy, how often are each of these enforcement activities used?

	Never Used	Rarely Used	Occa-sionally Used	Fre-quently Used	Don't know
Violations documented in tenant record					
Warning letters issued					
Fines imposed					
Meetings held with ten-ant					
Other (please specify)					

Does your PHA track violations?

- ◯ Yes
- ◯ No
- ◯ Don't know

Does your PHA track subsequent actions against violations?

- ◯ Yes
- ◯ No
- ◯ Don't know

How does your PHA learn about violations? Check all that apply.

- ☐ Residents
- ☐ Security/Housing police
- ☐ Maintenance Staff
- ☐ Other housing authority staff
- ☐ Other (Please Specify): ______________________________
- ☐ Don't know

With regard to the smoke-free policy:

In the past year, how many households have been reported for violations?

____

In the past year, how many violations made their way to housing court?

____

In the past year, how many violations have resulted in evictions?

____

Did you participate in any technical assistance or training provided by HUD or other organizations (for example, American Lung Association, American for non-Smokers Rights, etc)?

- ◯ Yes
- ◯ No
- ◯ Don't know

How helpful was the technical assistance/training?

- ◯ Unhelpful
- ◯ Somewhat unhelpful
- ◯ Neither unhelpful nor helpful
- ◯ Somewhat helpful
- ◯ Helpful
- ◯ Don't know

Our last set of questions is about your successes, challenges, and any lessons to be shared with other PHAs as they begin to implement smoke-free policies.

Has your policy changed after it was first adopted?

- ◯ Yes, please explain: ______________________________
- ◯ No
- ◯ Don't know

Is there anything you feel you did particularly well in planning and implementing the smoke-free policy?

____
____
____
____
____

What advice would you offer to PHAs just beginning to develop their smoke-free policy?

____
____
____
____
____

Thank you for your time.
